# Neuron-specific enolase in patients with acute and chronic schizophrenia, diversity of approaches: marker of neuronal death, neurodegeneration or neurodevelopmental theory in schizophrenia? a single-center case-control study

**DOI:** 10.3389/fpsyt.2025.1520192

**Published:** 2025-05-06

**Authors:** Alicja Sierakowska, Ewa Niewiadomska, Sebastian Łabuda, Anna Bieniasiewicz, Mateusz Roszak, Beata Łabuz-Roszak

**Affiliations:** ^1^ Department of Family Medicine and Public Health, Institute of Medical Sciences, Faculty of Medicine, University of Opole, Opole, Poland; ^2^ Department of Psychiatry, St. Jadwiga Regional Specialized Hospital, Opole, Poland; ^3^ Department of Epidemiology and Biostatistics, Faculty of Public Health in Bytom, Medical University of Silesia, Katowice, Poland; ^4^ Department of Neurology, St. Jadwiga Regional Specialized Hospital, Institute of Medical Sciences, University of Opole, Opole, Poland; ^5^ Student Scientific Association at the Department of Neurology, Institute of Medical Sciences, University of Opole, Opole, Poland

**Keywords:** neuron-specific enolase, schizophrenia, biomarker, positive symptoms, negative symptoms

## Abstract

**Introduction:**

Schizophrenia is a mental illness that affects a diverse group of patients, but the underlying causes of the symptoms can vary. There is currently a lot of research being done on the use of biomarkers in the diagnosis of schizophrenia, including neuron-specific enolase (NSE). The aim of this study was to evaluate and compare NSE concentrations in the serum of patients with schizophrenia and to determine possible relationships between NSE and the duration and severity of positive and negative symptoms of schizophrenia.

**Materials and methods:**

The study included 59 patients with schizophrenia and 60 healthy controls. NSE serum concentrations were assessed in all subjects. The Scale for the Assessment of the Negative Symptoms (SANS) and the Scale for the Assessment of the Positive Symptoms (SAPS) were used to assess the symptoms of schizophrenia.

**Results:**

The mean serum concentration of NSE in patients with schizophrenia was statistically significantly lower than in healthy controls. A weak negative correlation between NSE levels and the SANS score and a weak positive correlation between NSE levels and the SAPS score were found, but the results were not statistically significant. No relationship was reported between age, sex, disease duration, dependence on others or laboratory findings and NSE levels.

**Discussion:**

The study found lower NSE levels in patients with schizophrenia. A tendency towards correlation between severity of negative symptoms of schizophrenia and decreased levels of NSE were observed. In addition, a trend was noted between an increase in NSE level and the severity of positive symptoms. The results of the experiment should be confirmed in further studies.

## 1 Introduction

It is impossible to explain the etiopathogenesis of schizophrenia without understanding the pathophysiology of psychotic symptoms. The term “schizophrenia” refers to a heterogeneous population of patients with similar symptoms. However, their background may be different (1). Identification of biological, neurobiological and genetic endophenotypes is necessary to search for objective markers of psychotic symptoms such as hallucinations or delusions (2, 3). The overall scope of clinical assessment of patients remains broad. Not only (positive and negative) symptoms should be considered, but also cognitive impairment, the level of social functioning, the mechanisms of excitation/regulation, the level of functioning of the brain network, physiology, behavior and self-assessment of patients and the relative impact of the disease on the patient’s life (4). Currently, many studies are conducted on the use of biomarkers in the diagnosis of schizophrenia. Among the substances, the following are distinguished: individual DNA methyltransferases (DNMTs) (5); modifications of histones (mainly H2, H3 and H4 nucleosomes) (6); non-coding RNAs (ncRNAs) (7); protein S100B (8) and neuron-specific enolase (NSE) (9). The NSE protein was discovered by Moore and McGregor in 1965 (10). Enolase isoenzymes in eukaryotic organisms include enolase-1 (α-enolase), enolase-2 (γ-enolase) and enolase-3 (β-enolase), which are encoded by the *Eno1*, *Eno2*, and *Eno3 genes* (11), respectively. Enolase isoforms are formed by homo- and heterodimers (αα, αβ, αγ, ββ, γγ) (12). Isoenzymes with a γ subunit are referred to as NSE (13), which is widely distributed in the central nervous system (CNS) (14). It also acts as a biomarker of neurodegenerative mechanisms and some neurological diseases. Different enzyme concentrations may indicate abnormalities in neurodevelopmental and neuroprotective processes (15). NSE has also been used to assess the extent of brain damage due to trauma or hypoxic-ischemic processes (16). Elevated NSE serum concentrations correlate with the occurrence of some cancers, mainly neuroendocrine tumors (NETs) and small cell lung carcinoma (SCLC) (17, 18). However, there are reports indicating different concentrations of the enzyme in mental disorders. Studies indicate decreased levels of NSE in patients with schizophrenia. However, no analyses have been conducted in terms of a possible correlation between the severity of positive and negative symptoms of schizophrenia and NSE. In addition, the impact of disease duration and patient functioning on potential changes in NSE levels has not been assessed yet.

Therefore, the aim of the study was to assess and compare NSE serum concentrations in patients with schizophrenia in relation to healthy controls and to determine possible relationships between NSE and the duration and the course of the disease, dependence on others and the severity of positive and negative symptoms of schizophrenia.

## 2 Materials and methods

### 2.1 Participants

Patients hospitalized in the Department of Psychiatry, St. Jadwiga Regional Specialized Hospital in Opole, Poland, from August 1^st^ to December 30^th^ 2023 were enrolled in the study. The patients were admitted to the hospital for an exacerbation of schizophrenic symptoms, as indicated by increasing delusions, perceptual disturbances usually in the form of auditory hallucinations or pseudo hallucinations. The inclusion criteria were as follows: diagnosis of paranoid or residual schizophrenia (F20.0, F20.5; ICD-10), age > 18 years and written informed consent to participate in the study. The exclusion criteria included past head injury (this information was obtained by interviewing the patient and his family, if there was a recent head CT scan, the authors reviewed the results before qualifying the patient for the study), cardiac arrest, myocardial infarction, stroke, neurodegenerative disease, epilepsy, cognitive impairment making it impossible to give informed consent, neuroendocrine tumors and lung cancer. Participation in the study was offered to 70 patients who met all the inclusion and exclusion criteria. However, informed consent was given by 59 patients who were finally enrolled in the study. The control group consisted of 60 healthy volunteers who were age-matched with patients and were hospital employees who agreed to participate in the study. Based on the history and medical records, the following were determined: demographic data (gender, age, education, years of education, professional activity) and clinical data of patients (disease duration, duration of exacerbations and remissions, drugs). Schizophrenia-related degree of disability was also assessed based on the patient’s ability to function independently, work and make decisions. Three categories were distinguished: completely independent, partially dependent and completely dependent.

### 2.2 Clinimetric examination

In the group of patients with schizophrenia, the severity of positive and negative symptoms of schizophrenia was measured using validated Polish questionnaires, i.e., The Scale for the Assessment of the Negative Symptoms (SANS) and the Scale for the Assessment of the Positive Symptoms (SAPS) (19, 20). The former scale includes five subscales, which include a total of 25 items. The subscales include: 1) affective blunting (SANS-I; 40 points), 2) alogia (SANS-II; 25 points), 3) avolition and apathy (SANS-III; 20 points), 4) anhedonia and asociality (SANS-IV; 25 points) and 5) attention (SANS-V; 15 points) (21, 22). The SAPS scale contains 34 items, forming four subscales: 1) hallucinations (SAPS-I; 35 points), 2) delusions (SAPS-II; 65 points), 3) bizarre behavior (SAPS-III; 25 points) and 4) positive formal thought disorder (SAPS-IV; 45 points) (23). The clinimetric examination was carried out independently, by two people: physicians, a psychiatric specialist and a psychiatry resident, after which the average of the results was drawn. All patients were examined only by the two designated persons to avoid error due to differences in interpretation of the severity of a given symptom. Scaling was done on the basis of the patient’s direct clinical examination. The authors decided to choose the SANS and SAPS scales because of their far greater accuracy, by assessing more parameters, compared to newer scales such as CAINS and BNSS, which provided the opportunity for a more accurate analysis of individual symptoms.

### 2.3 NSE measurement

Venous blood (10 ml) was collected into a tube with a clotting activator between the first and 14th day of hospitalization. Blood was centrifuged and serum was obtained. The determination of NSE levels was performed using chemiluminescence immunoassay (CLIA) (Snibe Maglumi 2000 plus) (normal range 0-15.7 ng/ml). In addition, basic laboratory parameters were determined (complete blood count, glucose, creatinine, sodium, potassium, alanine aminotransferase, aspartic aminotransferase, thyroid-stimulating hormone levels).

The study was approved by the Bioethics Committee (No UO/0016/KB/2023).

### 2.4 Statistical analysis

MS Excel 2019 (Microsoft Office) was used to collect the data and Statistica 13.3 (StatSoft Poland) was used for calculations. Qualitative data were presented as numbers and percentages [n (%)]. Quantitative data were presented as the mean and standard deviation (X ± SD) and the median and quartiles [M (Q1-Q3)]. The Shapiro-Wilk test was used to assess the compliance of the data with the normal distribution. In the case of asymmetric distributions, the Mann-Whitney U test was applied to assess the significance of differences in two groups, and the Kruskal-Wallis test was used to assess the significance of differences in three or more groups. The Spearman correlation coefficient was used with the correlation coefficient significance test to evaluate the strength of the relationship of the features deviating from the normal distribution. Statistical significance was adopted at p<0.05.

## 3 Results

The study group consisted of 59 patients with schizophrenia, including 30 women (50.9%) and 29 men (49.2%). The mean age of the patients was 44.4 ± 15.4 years (range 20-81 years). The control group included 60 healthy subjects, i.e., 43 women (71.7%) and 17 men (28.3%). The mean age was 38.0 ± 15.8 years and did not differ statistically significantly from the study group.

In patients with schizophrenia, the mean education period was 12.4 ± 2.9 years (median 12; range 11-13 years). The study participants graduated from special needs school or had primary education (n=15, 25.4%), vocational education (n=15, 25.4%), secondary vocational (n=13, 22%), secondary (n=8, 13.6%) and higher education (n=8, 13.6%). Only ten patients (16.9%) were employed, while 36 subjects (61%) were not professionally active (28 received pension/retirement benefits, and the rest were unemployed). The controls had higher (n=31, 51.6%) or secondary education (n=29, 48.2%). All of them were professionally active and their work corresponded to their education.

In the study group, the mean duration of disease (from diagnosis) was 16.3 ± 14.2 years (median 13; 7-23 years). The mean time from the first symptoms of schizophrenia was 16.8 ± 14.3 years (median 15; 7-23 years). The mean longest exacerbation treated in hospital was 6.1 ± 42.8 days (median 56.5; 34-82 days), while the longest remission was 4.7 ± 3.7 years (median 4; 2-6.2 years). Almost all patients with schizophrenia (except for one subject) were on drugs during the study and clozapine and olanzapine were the most common (**Supplementary Material Table 1**).

Mean levels of laboratory parameters in patients with schizophrenia are given in **Table 1**. **Table 2** shows the results of the severity of positive symptoms (SAPS) and negative symptoms (SANS) of schizophrenia. Significantly higher scores were obtained by women in the combined SANS+SAPS, SANS IV and total SAPS and SAPS IV scores.

**Table 1:** The mean SANS/SAPS levels in patients with schizophrenia (total and by gender).

SANS / SAPS	Max Score	Total		Women		Men		p-value^A^
		X ± SD	M (Q1-Q3)	X ± SD	M (Q1-Q3)	X ± SD	M (Q1-Q3)	
SANS + SAPS	295	89.4 ± 34.8	86 (63-114)	98.9 ± 33.1	91 (70-128)	79.6 ± 34.3	68 (60-98)	0.030^A^
SANS – total	125	56.6 ± 20.1	59 (40-73)	61.5 ± 17.4	60.5 (49-74)	51.6 ± 21.8	52 (36-66)	0.060^A^
SANS – I	40	22.4 ± 5.7	23 (18-27)	23.3 ± 5.2	24 (19-27)	21.5 ± 6.2	22 (15-26)	0.230^A^
SANS - II	25	8.6 ± 5.1	8 (5-12)	9.1 ± 4.8	9 (6-12)	8 ± 5.4	6 (4-12)	0.410^A^
SANS – III	20	7.7 ± 4.5	8 (4-11)	8.7 ± 4.5	8.5 (6-12)	6.6 ± 4.4	6 (3-10)	0.080^A^
SANS – IV	25	12.5 ± 5.9	13 (10-17)	14.2 ± 4.5	14 (11-17)	10.7 ± 6.6	12 (6-15)	0.020^A^
SANS – V	15	5.5 ± 3.5	6 (3-8)	6.2 ± 3.5	6 (4-9)	4.9 ± 3.4	4 (3-8)	0.160^A^
SAPS – total	170	32.7 ± 21.7	29 (15-50)	37.8 ± 20.8	34 (24-50)	27.6 ± 21.7	21 (13-39)	0.040^B^
SAPS - I	35	5.9 ± 6.6	3 (0-12)	6.2 ± 6.9	3 (0-13)	5.6 ± 6.3	2 (0-11)	0.740^B^
SAPS - II	65	9.6 ± 8.8	8 (3-14)	10.8 ± 8.9	8 (6-14)	8.3 ± 8.8	6 (0-13)	0.170^B^
SAPS - III	25	5.8 ± 2.9	6 (3-7)	5.9 ± 3.1	6 (3-7)	5.7 ± 2.8	6 (3-8)	0.960^B^
SAPS - IV	45	11.8 ± 11.2	7 (3-20)	14.9 ± 11.3	13.5 (6-26)	8.7 ± 10.3	5 (2-9)	0.020^B^

Quantitative data presented as mean and standard deviation (X ± SD) and median with quartiles – M (Q1-Q3); Maximum score of SANS/SAPS (Max); p-value A, the Student’s t-test result; p-value B, the Mann-Whitney U test result;

SANS, Scale for the assessment of the negative symptoms.

SAPS, Scale for the assessment of the positive symptoms.

SANS-I, Scale for the assessment of the negative symptoms (AFFECTIVE FLATTENING OR BLUNTING).

SANS-II, Scale for the assessment of the negative symptoms (ALOGIA).

SANS-III, Scale for the assessment of the negative symptoms (AVOLITION/APATHY).

SANS-IV, Scale for the assessment of the negative symptoms (ANHEDONIA/ASOCIALITY).

SANS-V, Scale for the assessment of the negative symptoms (ATTENTION).

SAPS -I, Scale for the assessment of the positive symptoms (HALLUCINATIONS).

SAPS-II, Scale for the assessment of the positive symptoms (DELUSIONS).

SAPS-III, Scale for the assessment of the positive symptoms (BIZARRE BEHAVIOR).

SAPS-IV, Scale for the assessment of the positive symptoms (POSITIVE FORMAL THOUGHT DISORDER).

**Table 2:** Correlations between SANS/SAPS and disease duration, the longest exacerbation and longest remission in the study group.

SANS / SAPS	Age [years]		Disease duration [years]		Longest exacerbation treated in hospital [days]		Longest remission [years]	
	R	p-value	R	p-value	R	p-value	R	p-value
SANS + SAPS	0.26	0.048	0.26	0.04	0.21	0.12	0.17	0.21
SANS – total	0.45	0.0004	0.37	0.004	0.37	0.004	0.17	0.20
SANS – I	0.27	0.04	0.24	0.07	0.28	0.03	0.17	0.20
SANS – II	0.37	0.004	0.43	0.0007	0.35	0.01	0.28	0.04
SANS – III	0.33	0.01	0.26	0.049	0.26	0.04	0.01	0.95
SANS – IV	0.53	0.00001	0.29	0.02	0.34	0.01	0.15	0.25
SANS – V	0.25	0.06	0.21	0.11	0.26	0.046	0.10	0.46
SAPS – total	-0.08	0.55	0.02	0.90	-0.01	0.95	0.05	0.74
SAPS – I	-0.38	0.003	-0.23	0.08	-0.08	0.56	-0.21	0.12
SAPS – II	-0.14	0.27	-0.10	0.47	-0.05	0.69	-0.04	0.75
SAPS – III	0.01	0.93	0.01	0.93	-0.05	0.69	-0.02	0.91
SAPS – IV	0.15	0.27	0.19	0.14	0.02	0.86	0.24	0.07

Data presented as R- Spearman’s R correlation coefficient; p-value – result of the significance test of the Spearman’s R correlation coefficient; Abbreviations as in **Table 3**.

We performed the analysis of the relationships between SANS and SAPS scores and the duration of the disease, the duration of exacerbations and remission (**Supplementary Material Table 2**). A significant positive correlation was noted between SANS (overall, in terms of alogia - speech disorders/logical thinking disorders, avolition/apathy, anhedonia – poor social contacts) and age, disease duration and the longest disease exacerbation. In addition, a significant negative correlation was observed between the occurrence of hallucinations and age.

**Figure 1:**
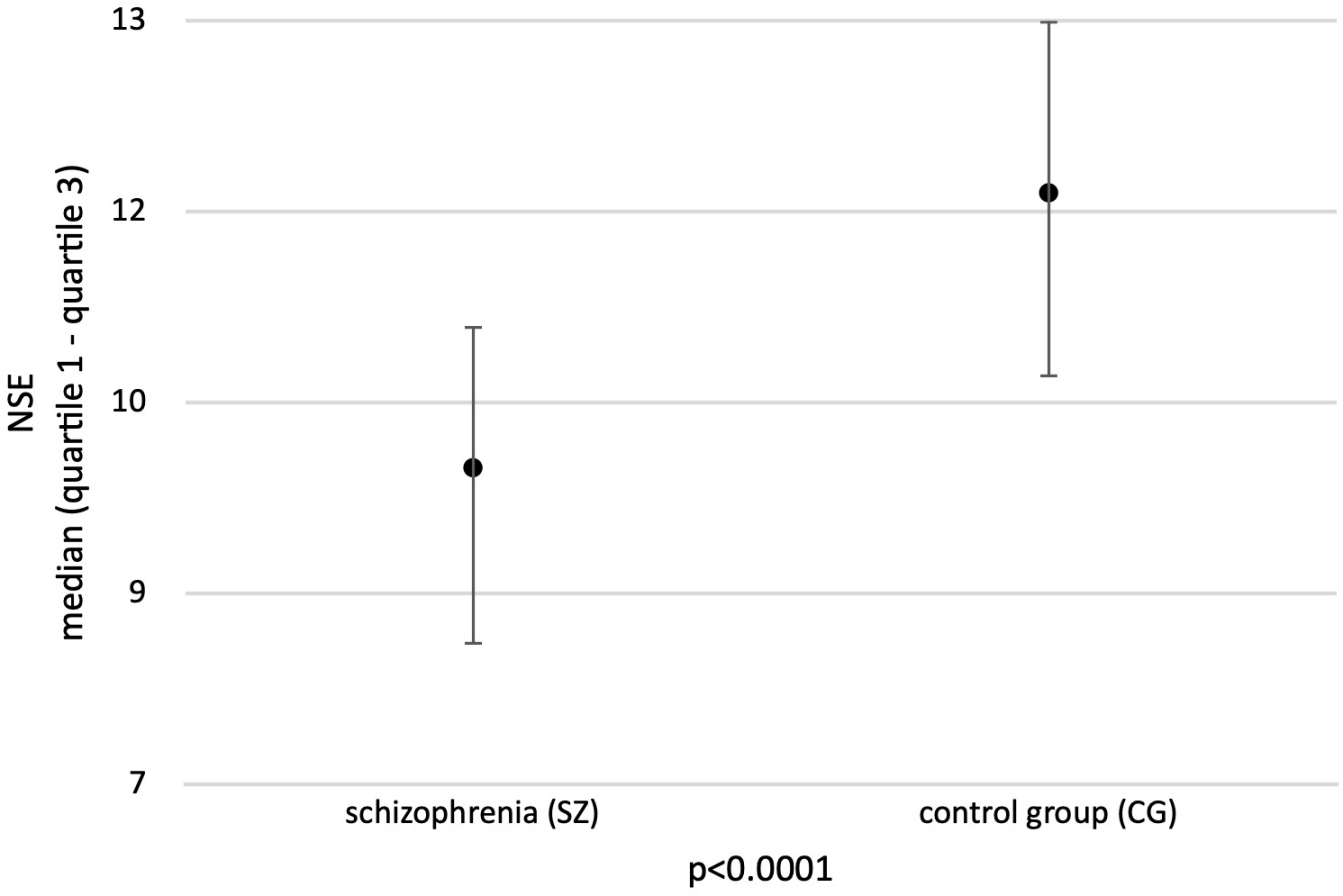
Mean concentration of NSE in the study groups. Adapted with permission from [Neuron-specific enolase as biomarker in schizophrenia] by [Sierakowska A., Niewiadomska E., Łabuda S., Roszak M., Bieniasiewicz A., Łabuz-Roszak B.], [Abstract book - 13th Student Congress of Neuroscience - NeuRi 2024 Rijeka - Rabl; SSN: 2623-6273].

The mean NSE level in the group of patients with schizophrenia was 9.5 ng/ml (quartile range 8.1- 10.6 ng/ml) and was statistically significantly lower than the mean NSE concentration in the control group (11.7 ng/ml; quartile range 10.2-13.0 ng/ml) (p<0.0001) (**Figure 1**). No statistically significant differences were found in mean NSE levels in both groups based on sex and age.

In the study group, two patients (2.5%) presented with abnormal NSE levels (>15.7 ng/ml) compared to one subject in the control group (1.7%) (**Table 3**).

**Table 3:** Characteristics of patients with schizophrenia with elevated NSE levels.

Patient characteristics	Patient 1	Patient 2
Age (years)	43	39
Sex	Woman	Woman
NSE level (ng/ml)	16.3	17.2
Disease duration [years]	20	0.5
Longest exacerbation (hospital stay) [days]	136	60
Longest remission [years]	8	0.2
Dependence range completely dependent: n=10, 16.9% partially dependent: n=28, 47.5% independent: n=21, 35.6%	completely dependent	partially dependent
Currently used antipsychotic drugs	Risperidone	Olanzapine
Previously used antipsychotic drugs	Olanzapine, flupentixol, haloperidol, risperidone, paliperidone, sulpiride, quetiapine, clozapine, levomepromazine	Olanzapine
SANS X ± SD: 56.6 ± 20.1 Max: 125	97	58
SAPS X ± SD: 32.7 ± 21.7 Max: 170	50	27

Dependence range: independent/ partially dependent/ completely dependent;

SANS, Scale for the assessment of the negative symptoms.

SAPS, Scale for the assessment of the positive symptoms.

**Figure 2:**
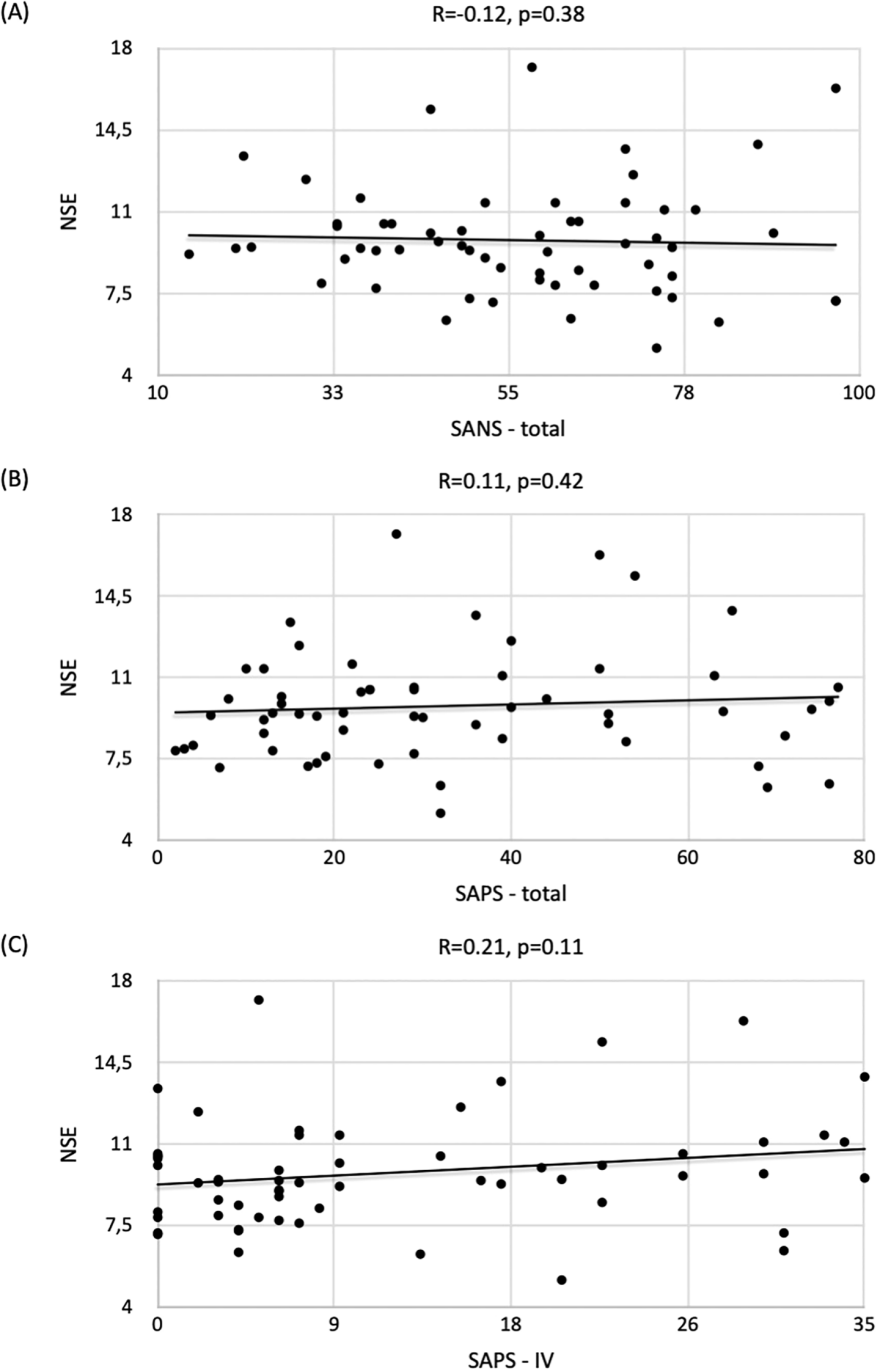
Correlation of NSE concentrations with respect to **(A)** SANS / **(B)** SAPS / **(C)** SAPS-IV scores and the strength of correlation expressed by the Spearman correlation coefficient (R) in the group of patients with schizophrenia.

A weak negative correlation was observed between NSE concentrations and the overall SANS score (R=-0.12, p=0.38) (**Figure 2A**) and a weak positive correlation was noted between NSE concentrations and the overall SAPS score (R=0.11, p=0.42) (**Figure 2B**), but the results did not reach statistical significance. In addition, a weak correlation was observed between SAPS IV and increasing NSE levels (R=0.21, p=0.11) (**Figure 2C**).

As for other SANS/SAPS scores, the correlation with NSE levels was statistically nonsignificant: SANS+SAPS (R=-0.04, p=0.77); SANS-I (R=-0.07, p=0.60); SANS-II (R=-0.10, p=0.47); SANS-III (R=-0.07, p=0.58); SANS-IV (R=-0.02, p=0.86); SANS-V (R=-0.09, p=0.51); SAPS-I (R=-0.01, p=0.94); SAPS-II (R=-0.01, p=0.93); SAPS-III (R=-0.03, p=0.82) and SAPS-IV (R=0.21, p=0.11).

No statistically significant relationship was found between laboratory findings and NSE levels in patients with schizophrenia. The values of Spearman’s R correlation coefficient with the result of the significance test were as follows: WBC (R=0.13, p=0.31); RBC (R=0.02, p=0.86); HGB (R=-0.09, p=0.51); MCV (R=-0.09, p=0.49); MCH (R=-0.04, p=0.76); PLT (R=0.1, p=0.46); PCT (R=0.09, p=0.52); glucose (R=-0.01, p=0.97); creatinine (R=-0.03, p=0.8); sodium (R=-0.05, p=0.7); potassium (R=-0.14, p=0.28); AST (R=-0.07, p=0.61); ALT (R=-0.11, p=0.4) and TSH (R=-0.06, p=0.67).

Additionally, no significant relationship was found between NSE levels and the time from the onset of symptoms (in years) (R=0.02, p=0.9), disease duration (in years) (R=0.02, p=0.87), the duration of the longest exacerbation (number of days of hospital stay) (R=0.02, p=0.9) and the time of the longest remission (in years) (R=0.01, p=0.96).

No significant differences were reported in the mean NSE levels in the groups based on dependence on others due to schizophrenia (p=0.93). In the subgroups according to the level of dependence, the following NSE levels were reported: independent - 9.5 ± 1.7 (M=9.4; range 8.3-10.2), partially dependent - 9.9 ± 2.5 (M=9.6; 8.5-10.8) and completely dependent - 10.1 ± 3.3 (M=9.4; 7.2-12.6). No analysis of the relationship between NSE levels and drugs was performed due to the small size of the groups (single patients).

## 4 Discussion

The study indicates significantly lower serum concentrations of NSE in patients with schizophrenia compared to the results of the controls, which is in line with most reports. The first report on NSE in patients with schizophrenia was published in 1991 (24). Serum NSE concentrations were assessed in 189 patients with schizophrenia and 112 controls. Patients with schizophrenia had lower concentrations of the biomarker compared to healthy controls. The results obtained in our study are also similar to those published by Liu et al., who assessed NSE concentrations in 43 patients with the first episode of schizophrenia (FES), 39 with chronic schizophrenia (CSZ) and 47 controls. The mean level of NSE in the CSZ group was statistically significantly lower compared to the FES group and controls. Andreou et al. also described similar findings. In a very large cohort (n=1132) with schizophrenia or bipolar spectrum disorder, they found significantly lower NSE levels in relation to the control group (19).

There are also reports with different study results. Steiner et al. assessed NSE levels in 12 patients with the first episode of schizophrenia and 17 controls and found no statistically significant differences in NSE levels between the groups. The discrepancy in the results could be caused by too small a sample size. Their results can be explained based on the pathophysiology of schizophrenia from the perspective of the theory of neurodevelopmental disease. NSE is involved in the differentiation, maturation and migration of neurons (15, 20). In embryonic brains from mammals and in neuronal cultures, it has been shown that low-differentiated neuronal cells primarily contain non-neuronal enolase (NNE), while a switch to NSE takes place during neuronal maturation and cell migration. Available reports suggest impaired neuronal maturation in both schizophrenia and bipolar affective disorder (19). In the prefrontal cortex (PFC) region of the brains of schizophrenia patients, the level of neuronal differentiation is reduced compared to healthy subjects (21). NSE is involved in neuronal differentiation (15). Both Bleuler and Andreasen described schizophrenia from the perspective of the necessity of the occurrence of primary axial symptoms that affected global functioning of patients (25–27). Neurodevelopmental abnormalities in schizophrenia start with the formation of neuronal networks (e.g., at the axonal level). However, in most cases, dysfunction occurs at the level of sending and receiving information in the area of synaptic connections (28) and abnormal nerve cell development is associated with lower NSE concentrations. Given the association of axial symptoms with low activity of the mesocortical pathway, which has its terminus precisely in the PFC area, where a reduction in the level of neuronal differentiation was observed (21), one should expect lower concentrations of NSE values, which may correlate with the level of severity of negative symptoms (SANS).

To the best of our knowledge, this study is the first in which the results of NSE concentrations were compared to the scores of the SANS and SAPS. First of all, it should be noted that there is a possibility of a potential type II statistical error due to the sample size, which is why, as has been repeatedly stated, the research needs to be expanded and repeated.

The only available study in correlation with the authors’ work relating to the severity of positive as well as negative symptoms (which was also measured using the PANSS scale) is from 2007. Its purpose was to determine the level of neuronal damage in patients suffering from treatment refractory schizophrenia (TRS) compared to those with a diagnosis of non-refractory schizophrenics (NRS) using quantitative analysis of lipid peroxidation and NSE. Thirteen participants each with a diagnosis of TRS and NSR were included in each study group, as well as 13 healthy volunteers were classified as controls. The results of NSE levels as byproducts of lipid peroxidation remained higher in patients from the TRS group compared to those from the NSR or non-clinical group. Moreover, the elevation of NSE levels correlated with the severity of negative symptoms and at the same time with higher scores on the SANS scale (29). The results presented here remain different from those of the present study, but it should be noted the relatively small group of patients included in the study, which may explain the discrepancies between the papers.

A weak negative correlation between NSE levels and SANS scores indicates a possible relationship with the severity of negative symptoms of schizophrenia. Since there are no similar papers for the comparison with our findings, and considering the common relationship between the development of schizophrenia, catatonia and autism spectrum disorder (ASD) based on the iron triangle theory (30) and the common negative symptoms of schizophrenia and ASD, such as apathy, anhedonia, aspontaneity, passivity, or limited affect modulation), we decided to focus on the reports on NSE in ASD. Wang et al. (31) compared 131 pairs of age- and sex-matched autistic and control subjects. Venous blood serum was collected from the study participants and the hypermethylation of the neuron-specific gene (*ENO2*) (also known as NSE) was assessed. The hypermethylation of *ENO2* was found in 14.5% of the samples from the study group, which reduced the mean *ENO2* RNA level by approximately 70% relative to that in the controls. Furthermore, the average level of ENO2 protein expression in 19 subjects from the study group (15.18 ± 3.51 μg/L) was about half of that in the controls (33.86 ± 8.16 μg/L).

Other studies, however, showed different results. Ayaydin et al. (32) assessed 43 patients with ASD and 41 healthy controls. Serum levels of NSE in patients with ASD were significantly higher in the study group compared to the controls. A similar relationship was reported by Stancioiu et al. (33) who evaluated 41 patients with ASD. Umbilical cord blood was collected from the study participants and NSE levels were determined. They were elevated in 40 of the 41 samples (97.5%).

Some reports do not indicate any of the above relationships. For instance, Esnafoglu et al. (34) assessed 35 patients with ASD and 31 controls. On the basis of the assessment of serum NSE concentrations, no statistically significant differences were obtained between the groups. Similar results were presented by Kartalci et al. (35). Their study included 35 patients with ASD and 35 controls.

Therefore, the results are inconclusive and further research is warranted. Such a large discrepancy in the results is most likely related to too small a sample size. Nevertheless, the study conducted on the largest cohort (Wang et al.) is in line with our findings. Therefore, common negative symptoms in schizophrenia and ASD and the same neurodevelopmental etiopathogenesis of the diseases may account for the correlations.

In our study, we also observed a positive trend in terms of the relationship between NSE concentrations and the severity of positive symptoms (i.e., the severity of schizophrenia exacerbation). This may indicate neuronal impairment due to an ongoing psychotic process. However, some authors perceive schizophrenia from the perspective of a neurodegenerative disease. Neurodegenerative causes could be associated with abnormal genetic programming, which is manifested by cognitive disorders, whose occurrence indicates neuronal destruction (36, 37).

Available studies indicate a reduction in the volume of white as well as gray matter in patients with schizophrenia (38); moreover, abnormalities also occur at the level of mitochondria and the metabolic processes taking place there (39). Structural changes have been confirmed by neuroimaging studies using computed tomography (CT) or magnetic resonance imaging (MRI) in numerous reports (40–42). The most notable changes involve reductions in the volume of the prefrontal cortex, superior temporal cortex and hippocampus (43). Recent meta-analyses suggest a significant association with the sequelae of psychotic disorders and at the same time positive symptoms as a result of traumatic brain injury, associated with neuronal death (44). As mentioned in the introduction - neuronal damage is associated with the release of NSE from nerve cells and an increase in the concentration of the substance’s values in the extracellular space (16), which may explain the correlation of the severity of positive symptoms (and at the same time higher scores on the SAPS scale) with the level of NSE.

To the best of our knowledge, our study is the first to analyze the relationship between the duration of the disease, the results of additional laboratory tests, functioning of patients with schizophrenia and NSE levels. Therefore, it is not possible to compare the results to the literature data and further research is warranted in this respect.

### 4.1 Limitations

The study group was relatively small and only patients with chronic schizophrenia were evaluated. In addition, the measurements were carried out at a single center and the patients were evaluated using SANS/SAPS by only two people, which could have led to potential bias. The change in NSE level over time was also not evaluated. Therefore, the results should be confirmed in multicenter studies on a larger cohort of patients with chronic schizophrenia and first-episode schizophrenia, taking into account the timing criteria of the measurements, preferably presenting a series of NSE measurements over a specific period of time. Such studies could also allow for the assessment of the impact of medication on NSE levels, which was not conducted in this study due to the small groups of patients taking the medication in question. The study did not take into account factors such as cigarette smoking, concomitant obesity or the type of diet patients follow. No reports were found that would relate to possible correlations between these factors and NSE concentration levels. Furthermore, it would be necessary to precisely determine the number of cigarettes smoked each day, the period of time the patient has been smoking, and the type of cigarettes (electronic, traditional, or tobacco lighters). Similar differentiation should be carried out in relation to the diet and potential excess weight. It also seems necessary to consider the possible correlation between the occurrence of the aforementioned factors as a direct result of the underlying disease (e.g. obesity as a consequence of long-term use of e.g. olanzapine; obesity, and often also co-occurring eating disorders as a consequence of impaired dopaminergic function, which underlies schizophrenia). These possible relationships could be the subject of separate studies, which nevertheless require further exploration in future work on NSE.

## 5 Conclusions

The study showed lower NSE levels in patients with schizophrenia. A tendency towards correlation between severity of negative symptoms of schizophrenia and decreased levels of NSE were observed. In addition, a trend was noted between an increase in NSE level and the severity of positive symptoms with formal thought disorder. No correlations were found between the duration of the disease, schizophrenia-induced dependence, or laboratory findings and serum NSE levels. Further studies are warranted to determine the relationships in a more representative group and to assess the dynamics of changes in NSE levels over time.

## Data Availability

The raw data supporting the conclusions of this article will be made available by the authors, without undue reservation.
