# Supplementary material for: Neuron-specific enolase in patients with acute and chronic schizophrenia, diversity of approaches: marker of neuronal death, neurodegeneration or neurodevelopmental theory in schizophrenia? a single-center case-control study
Source: Front Psychiatry. 2025 May 6;16:1520192. doi: 10.3389/fpsyt.2025.1520192 (PMC12089130; doi:10.3389/fpsyt.2025.1520192)
Supplement: Supplementary file 2 [file DataSheet2.pdf]

Table 1. The number and the list of drugs taken by patients with schizophrenia

Drugs		Currently	Previously (total amount of drugs used so far)
Number of drugs	0	1 (1.7%)	3 (5.1%)
	1	23 (39%)	7 (11.9%)
	2	26 (44.1%)	6 (10.2%)
	3	7 (11.9%)	10 (16.9%)
	4	1 (1.7%)	3 (5.1%)
	5	0 (0%)	7 (11.9%)
	6	1 (1.7%)	7 (11.9%)
	7	0 (0%)	8 (13.6%)
	8	0 (0%)	4 (6.8%)
	9	0 (0%)	3 (5.1%)
	11	0 (0%)	1 (1.7%)
Drug name	Alprazolam	0 (0%)	3 (5.1%)
	Amisulpride	9 (15.3%)	25 (42.4%)
	Aripiprazole	5 (8.5%)	10 (16.9%)
	Chlorprothixen	6 (10.2%)	15 (25.4%)
	Diazepam	0 (0%)	21 (35.6%)
	Flupentixol	2 (3.4%)	13 (22%)
	Haloperidol	3 (5.1%)	24 (40.7%)
	Cariprazine	5 (8.5%)	4 (6.8%)
	Clonazepam	0 (0%)	16 (27.1%)
	Clozapine	13 (22%)	21 (35.6%)
	Quetiapine	6 (10.2%)	21 (35.6%)
	Levomepromazine	3 (5.1%)	19 (32.2%)
	Lorazepam	0 (0%)	40 (67.8%)
	Lurasidone	4 (6.8%)	6 (10.2%)
	Olanzapine	20 (33.9%)	41 (69.5%)
	Paliperidone	1 (1.7%)	4 (6.8%)
	Promazine	3 (5.1%)	21 (35.6%)
	Risperidone	8 (13.6%)	24 (40.7%)

Sulpiride	0 (0%)	7 (11.9%)
Zuclopenthixol	2 (3.4%)	11 (18.6%)

Data presented as numbers and percentages – n (%)

Table 2. The mean levels of the basic laboratory parameters of patients with schizophrenia

Parameters	Concentration X ± SD	Range Min-Max
WBC [1000/ul]	8.09 ± 5.23	4.3-10
RBC (mln/ul)	4.58 ± 0.54	3.7-5.1
Hemoglobin (g/dl)	14.16 ± 1.74	12-18
MCV (fL)	91.38 ± 5.34	77-94
MCH (pg)	31.06 ± 2.23	27-34
Platelets (G/l)	249.31 ± 62.35	150-400
PCT (ng/ml)	0.251 ± 0.06	0.17-0.38
Glucose (mg/dl)	98.85 ± 14.35	61.5-99.5
Creatinine (mg/dl)	0.79 ± 0.15	0.5-1.2
Sodium (mmol/l)	140.78 ± 2.84	134-145
Potassium (mmol/l)	4.29 ± 0.29	3.5-5.1
AST (U/l)	21.27 ± 9.79	0-38
ALT (U/l)	25.46 ± 22.69	0-41
TSH (uIU/ml)	2.18 ± 1.93	0.27-4.20

Quantitative data presented as mean and standard deviation (X ± SD);
Laboratory norms (Min-Max);
MCV - mean corpuscular volume
MCH - mean corpuscular hemoglobin
PCT - procalcitonin
ALT - alanine aminotransferase
AST - asparagine aminotransferase
TSH - thyroid-stimulating hormone
